# The impact of water depth and speed on muscle fiber activation of healthy dogs walking in a water treadmill

**DOI:** 10.1186/s13028-021-00612-z

**Published:** 2021-11-24

**Authors:** Anne Désiré Vitger, Tanja Bruhn-Rasmussen, Eja Oppenlænder Pedersen, Lene Høeg Fuglsang-Damgaard, Adrian Paul Harrison

**Affiliations:** 1 Department of Veterinary Clinical Sciences, Faculty of Health and Medical Sciences, University of Copenhagen, Dyrlægevej 16, 1870 Frederiksberg C, Denmark; 2 Herfølge Dyreklinik, Vordingborgvej 78 A, 4681 Herfølge, Denmark; 3 Husum Dyreklinik, Frederikssundsvej 326, 2700 Brønshøj, Denmark; 4 Department of Animal Science, Aarhus University, Blichers Alle 20, 8830 Tjele, Denmark; 5 Department of Veterinary and Animal Sciences, Faculty of Health and Medical Sciences, University of Copenhagen, Grønnegårdsvej 15, 1870 Frederiksberg, Denmark

**Keywords:** Acoustic myography, AMG, Rehabilitation, Canine, Skeletal muscle, Hydrotherapy

## Abstract

**Background:**

Water treadmills are frequently used in the rehabilitation of dogs, for example with the purpose of re-building muscular strength after surgery. However, little is known about how different water depths and velocities affect the muscular workload during aquatic locomotion. This study used acoustic myography to assess hind limb muscle fiber activation in 25 healthy large-breed dogs walking in a water treadmill. Acoustic myography sensors were attached to the skin over the vastus lateralis of the quadriceps and the biceps femoris muscles. The dogs walked at two velocities (30 and 50 m/min) and four water depths: bottom of the pads, hock, stifle and mid-femur. Acoustic myograph signals were analyzed for changes in three muscle function parameters: efficiency/coordination (E-score) and spatial (S-score) and temporal (T-score) summation.

**Results:**

Differences between E, S, and T were statistically significant compared across different speeds (30, 50) and water levels (0, 1, 2, 3) using a one-way ANOVA with multiple comparisons (Tukey; Geisser-Greenhouse correction) as well as a two-tailed one sample t-test. At 50 m/min in water at the mid-femur, the biceps femoris was less efficient (P < 0.001) and recruited more fibers (P = 0.01) at a higher firing rate (P = 0.03) compared to working in shallower water, while the vastus lateralis was also less efficient (P < 0.01), but spatial and temporal summation did not change significantly. At 30 m/min, biceps efficiency was reduced (P < 0.01) when water was at the mid-femur compared to the bottom of the pads level. Walking in stifle- or hock-deep water did not show increased muscle activation for either muscle compared to walking in water at the bottom of the pads.

**Conclusion:**

More muscle activation was required to walk in water at a depth at the level of the mid-femur compared to shallower water, and this exercise was more demanding for the biceps femoris, a muscle engaged in propulsion, than for vastus lateralis. These findings may help practitioners towards making more precise rehabilitation protocols.

## Background

The water treadmill is a popular tool in canine rehabilitation and conditioning. Due to the buoyancy of water, joint load is reduced when a subject is submerged, and due to the viscosity of water, moving through the resistance of water requires more muscle work than is required for moving through air. Thus, when moving in water at different water levels, muscle fiber activation will be influenced by any alteration in buoyancy and resistance. Yet despite this, little is known about the net result of these factors on the muscular workload. Increasing water depth will increase the resistance that the moving body has to overcome, and therefore, it is intuitively logical that the workload of movement will increase with increasing water depth. The effect may, however, be counteracted by a decrease in weightbearing forces due to buoyancy. Studies of horses walking in a water treadmill showed that heart rate did not increase with increasing water depth [1, 2], and these findings have led to the assumption, that higher water levels do not constitute a more strenuous exercise than shallower water levels [3]. This assumption was supported by the results of a recent study using surface electromyography (sEMG) to evaluate muscular activity of dogs during water walking [4]. However, oxygen consumption has been shown to increase with rising water levels up to the mid-femur level in humans and horses [5, 6]. With this discrepancy in the relevant research, it is clear that more studies on the subject are required to enable canine rehabilitation therapists to design evidence-based protocols for muscle strengthening in water.

This study evaluates the activity of two major hind limb muscles with different actions: the biceps femoris (BF) being a muscle primarily engaged in propulsion, and the vastus lateralis of the quadriceps femoris muscle (QVL) being a protractor and an important weightbearing muscle. Acoustic myography (AMG) was used to record muscular activity, while the dogs walked in a water treadmill. Through sensors placed on the skin with self-adhesive tape, AMG uses the vibrations of working muscles as a non-invasive and easy-to-use method to assess muscle activity, as shown in several recent studies involving human subjects [7, 8], horses [9, 10] and dogs [11, 12].

From the sound wave file elicited by the contracting muscles, three AMG parameters were obtained and scored from 0 to 10: E-, S- and T-scores. The E stands for efficiency. It is a measure of the duration of muscle fiber activity in relation to inactivity, and thus this score describes the efficiency or coordination of the muscular work. A well-coordinated muscle that is efficient in turning on and off will yield a high E-score. The S stands for spatial summation, or amplitude of the signal. If the amplitude is low, few muscle fibers are recruited, and the S-score will be high. The T stands for temporal summation, or frequency of muscle fiber recruitment. If the frequency with which muscle fibers are recruited is low, the T-score will be high. On the other hand, during hard work muscles may react by increasing fiber activity time as well as by recruiting more fibers at a higher frequency, and hence the E-, S- and T-scores will be low. For more details about AMG, the reader is referred to Fuglsang-Damgaard et al. [12].

This study evaluates the work of the BF and the QVL of healthy dogs walking in a water treadmill at two speeds and at four different water levels. The following hypotheses were tested: 1) The E-, S- and T-scores for both BF and QVL decrease with increasing speed, and 2) The E-, S- and T-scores for both BF and QVL decrease with higher water levels.

## Methods

The study was designed as a controlled clinical trial.

### Animals

Dogs were recruited via an advertisement on the social media Facebook. Apart from a presumed willingness to move in water, inclusion criteria were height between 50 and 75 cm at the withers, age between 1.5 and 8.0 years old, body condition score of 4–6/9 [13], good health with no known gait abnormalities and no history of serious musculoskeletal injury or disease. Prior to the procedure the owners gave a signed consent for their dog’s participation in the study, but their rights were reserved to stop the procedure at any point, if they found their dog to be stressed or uncomfortable.

### Preparation

Upon arrival at the Companion Animal Hospital, the dog was given 10 min to get acquainted with the surroundings, while the owners filled out a questionnaire about their dog’s data. Subsequently, a gait analysis as well as a palpatory examination for muscle symmetry and tone were performed by an experienced clinician. If no abnormalities were found, the dog was taken for a short walk in the water treadmill (Hydro Physio HP200, Shorline, Kansas City, KS, USA), so that it could get accustomed to the belt moving at the two speeds used in the trial (30 m/min and 50 m/min) but with just a few millimeters of water covering the belt of the treadmill (water level 0).

### Acoustic myography

**Fig. 1:**
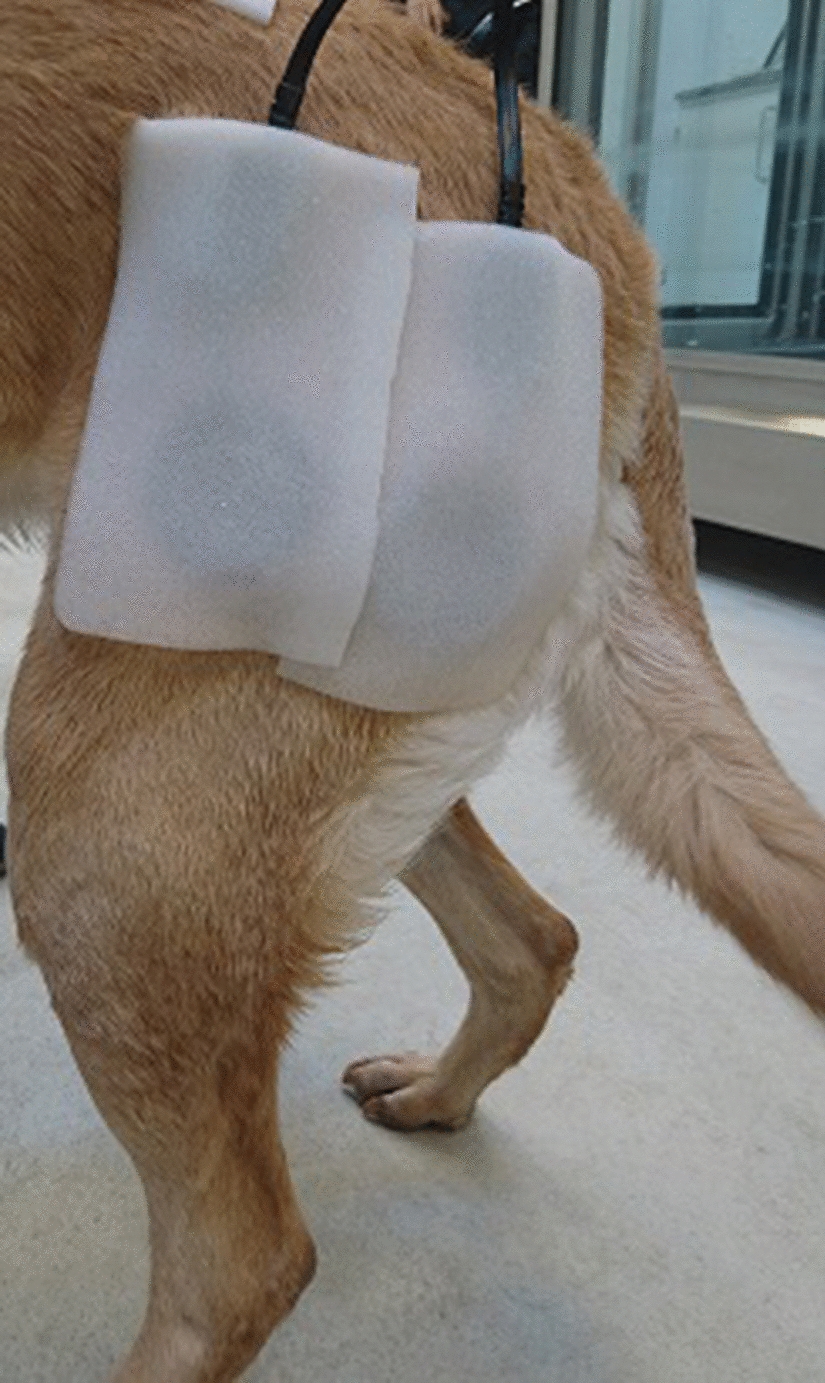
The position of acoustic myography sensors placed over the two muscle groups: *m. biceps femoris* (right) and *m. vastus lateralis of m. quadriceps* (left)

Outside the treadmill, the dog had the AMG sensors (CURO-diagnostic ApS, Bagsværd, Denmark) fitted. The following procedure was always performed by the same person (EOP). The muscle bellies of the BF and QVL were palpated, and the sites for sensor application were located on the same horizontal line slightly above the mid-femur. This step was supervised by a veterinary surgeon experienced in canine physiotherapy (ADV). The sites were prepared with acoustic gel (CURO-diagnostics ApS) which was thoroughly rubbed into the overlying hair to ensure a good connection with the skin above the BF and the QVL. The sensors (diameter of 50 mm) were also prepared with acoustic gel, before they were attached to the dog using flexible self-adhesive bandage (Animal Polster, Snögg Industry AS, Kristiansand, Norway) (Fig. 1). The sensors were then connected by cord to the hand-held AMG recording unit (CURO-diagnostics ApS, Bagsværd, Denmark). When the dogs walked in the water treadmill, recordings were made in the form of a WAV file to the CURO unit, and by wireless connection to an iPad, so that the recordings could be monitored in real-time. Data collection used a sampling frequency of 2000 Hz. After the trial, when the bandage was removed, it was checked that the gel was still present between the skin and the sensors.

### Trial protocol

During the trial the owner would stand in front of the dog holding the leash, while the person holding the CURO would be on the right side of the dog, and if in need of assistance a helper would be positioned on the left side of the dog. Before recordings in the water treadmill were initiated, the signal was tested by direct reading on an iPad, always by the same person (TBR). The dogs would walk at a slow walking speed (30 m/min) and at a moderately fast walking speed (50 m/min) in water at four different levels: Level 0, water to the bottom of the pads (to cover the belt, as dry running of the belt is not recommended). Level 1, water to the tibio-tarsal joint. Level 2, water to the tibial plateau. Level 3, water to the mid-femur. After each water level sequence, the belt was stopped for a short time until the tank was filled to the next level. All dogs completed the trial in the same order starting with level 0. Measurements at level 0 were repeated at the end of the trial to determine whether fatigue had occurred. Measurements were obtained when the person controlling the CURO found, by visual inspection, that the dog walked with a natural locomotion pattern for a continued period of at least 10 s, and when real-time recordings on an iPad showed a steady pattern for both muscle pairs. At this time a sequence of approximately 30 s was recorded for analysis. Care was taken to avoid any disturbances in the room during recordings. Water temperature was 27 °C.

### AMG data processing and analysis

Bilateral recordings from both muscles at two speeds and five water level sequences were stored on the CURO. Data were analyzed for efficiency (E-score), amplitude (S-score; spatial summation) and frequency (T-score; temporal summation), using the CURO System Software (CURO-diagnostics ApS, Bagsværd, DK). The analysis was carried out with a maximum frequency (max T) of 120 Hz and a maximum amplitude of 0.99 (max S) equivalent to approx. 1 V.

### Statistical analysis

All statistics were performed using GraphPad InStat 3 for Mac (Version 3.0b, 2003; Graph- Pad Inc., La Jolla, CA, USA). Every data column was initially tested for normality using a Shapiro–Wilk test, which works best when values are unique (we considered each dog in this study to be unique). The data was found to be parametric so column comparisons for muscle AMG parameters (E, S, T) at different speeds (30, 50) and water levels (0, 1, 2, 3) were checked for statistical significance using a one-way ANOVA with multiple comparisons (Tukey´s is best suited when comparing every mean with every other mean). Here an alpha threshold of 0.05 (95% CI) was selected and a Geisser-Greenhouse correction was performed since the data showed no signs of fatigue and so the sphericity assumption was considered not to have been violated (level 0_start_ compared to level 0_end_). Specific selected column comparisons using a two-tailed one sample t-test were also performed, for example changes in the E-score over time (0_start_ vs. 0_end_), as well as E-, S- and T-score comparisons at different speeds (30 vs 50 m/min), and E- S- and T-score comparisons at different water levels (0 vs 1, 1 vs 2, 1 vs 3, and 2 vs 3). Differences between means with a P value > 0.05 were considered non-significant. Values are presented as the mean ± the standard deviation*.*

## Results

25 dogs were included in the study and all completed the trial. Descriptive data are provided in Table 1.

**Table 1:** Study population

Breeds	Mixed breed (6), German Shepherd (6), Labrador Retriever (2), Border Collie, Dobermann, English Springer Spaniel, Malinois, Saluki, Swiss Shepherd
Age (years)^a^	5.1 ± 1.8
Sex	
Male	9 (3 neutered)
Female	16 (8 neutered)
Height (cm)^a^	59.0 ± 6.1
Body weight (kg)^a^	31.4 ± 7.5
Body condition score^b^	5 (4 – 6)

Descriptive data of 25 dogs who had acoustic myography measurements of *m. biceps femoris* and *vastus lateralis of m. quadriceps* while walking in a water treadmill at four different water levels and two velocities

^a^Mean value ± standard deviation, ^b^Median value (range)

Data for analysis were E-, S- and T-scores from bilateral recordings of the two muscles at two speeds and at four different water levels, and a repeat recording at water level 0 at the end of the trial. As no differences were found between the left and the right side, the bilateral data were pooled. Due to technical errors, data were excluded from BF of one dog and from QVL of another. Data from water level 3 of one dog were excluded due to swimming. Mean scores and standard deviations are shown in Table 2.

**Table 2:** Mean scores and standard deviations related to muscle, speed and water level

	Level 0	Level 1	Level 2	Level 3	Level 0_end_^a^
BF 30 m/min					
E	7.1 ± 2.0	7.1 ± 1.7	7.5 ± 1.8	6.4 ± 1.9	7.6 ± 1.6
S	8.1 ± 0.8	7.9 ± 1.0	8.2 ± 0.8	7.9 ± 0.8	8.1 ± 0.7
T	6.6 ± 2.2	7.4 ± 1.6	7.8 ± 1.5	6.5 ± 2.2	6.9 ± 2.3
BF 50 m/min					
E	5.4 ± 2.2	5.2 ± 2.1	5.1 ± 2.3	3.7 ± 2.5	5.5 ± 2.7
S	7.5 ± 1.4	7.6 ± 1.2	7.4 ± 1.3	7.1 ± 1.2	7.6 ± 1.2
T	6.1 ± 2.1	6.3 ± 2.1	6.9 ± 1.6	5.3 ± 2.3	6.5 ± 2.3
QVL 30 m/min					
E	7.3 ± 1.6	7.5 ± 1.9	7.7 ± 1.6	7.2 ± 1.9	7.8 ± 1.7
S	7.9 ± 0.9	7.9 ± 0.8	7.8 ± 1.0	8.1 ± 0.9	8.0 ± 0.8
T	7.2 ± 1.4	7.4 ± 1.3	7.9 ± 1.3	7.4 ± 1.4	7.4 ± 1.7
QVL 50 m/min					
E	5.8 ± 2.1	5.6 ± 2.0	5.5 ± 2.6	4.7 ± 2.6	6.2 ± 2.0
S	7.5 ± 1.3	7.3 ± 1.2	7.4 ± 1.2	7.5 ± 1.2	7.6 ± 1.0
T	6.9 ± 1.3	6.8 ± 2.0	7.1 ± 1.7	6.5 ± 2.0	7.3 ± 1.3

Acoustic myography from right and left *m. biceps femoris (BF)* and *vastus lateralis of m. quadriceps femoris (QVL)* on 25 large-breed dogs walking in water at two velocities (30 and 50 m/min) and at the following water levels: Level 0, at the bottom of the pad; level 1, at the tibio-tarsal joint; level 2, at the tibial plateau; level 4, at the mid-femur. Data were collected for muscle fiber resting time/total time (E-score), spatial summation (S-score), temporal summation (T-score), and scored from 1 to 10, where high scores indicate least muscle fiber activation

^a^Measurements at level 0 were repeated at the end of the trial

### Level 0_start_ compared to level 0_end_

There was no sign of muscles working harder when the first walk at water level 0 was repeated at the end of the trial. On the contrary, at the slow walking speed (30 m/min), the E-score (efficiency) increased with time in both BF (7%, P < 0.01) and QVL (6%, P = 0.01). At the faster walking speed (50 m/min) a reduction in firing rate (increased T-score) was seen at the end of the trial for QVL (6%, P = 0.02).

### Slow walk compared to fast walk

As shown in Table 3, increasing speed when walking at any depth of water (level 0,1, 2 and 3) required more muscle fiber activation for both muscles, as shown by lower E-scores (P < 0.001) as well as lower S- and T-scores (P < 0.05).

**Table 3:** Decrease in muscle function scores with increased speed

Score	Level 0 (bottom of pads)				Level 1 (hock)				Level 2 (stifle joint)				Level 3 (mid-femur)			
	BF	P*	QVL	P*	BF	P*	QVL	P*	BF	P*	QVL	P*	BF	P*	QVL	P*
E	24%	+ + +	21%	+ + +	27%	+ + +	25%	+ + +	32%	+ + +	29%	+ + +	42%	+ + +	35%	+ + +
S	7%	+ + +	5%	+ +	4%	+	8%	+ + +	10%	+ + +	5%	+	10%	+ + +	7%	+ + +
T	8%	+	4%	+	15%	+ + +	8%	+ + +	12%	+ + +	10%	+ +	18%	+ +	12%	+ +

Figures show the percentage decrease in fiber resting time/total time (E-score), spatial summation (S-score), temporal summation (T-score) of *m. biceps femoris* (BF) and *vastus lateralis of m. quadriceps* (QVL) when speed is increased from 30 m/min to 50 m/min. Measurements were obtained from 25 dogs walking in a water treadmill at four water levels. * P < 0.05 =  + , P < 0.01 =  +  + , P < 0.001 =  +  +  +

### Effects of water level at 30 m/min

The changes in E-, S- and T-scores that occurred with changing water levels at a slow walk are depicted in Table 4. E-scores for BF decreased when walking in water level 3 compared to shallower water depths. This change was also seen for QVL but only when compared to water at the stifle joint (level 2). When walking in water at level 1 or 2, both muscles increased their T-scores (firing rate decreased) compared to water level 0.

**Table 4:**
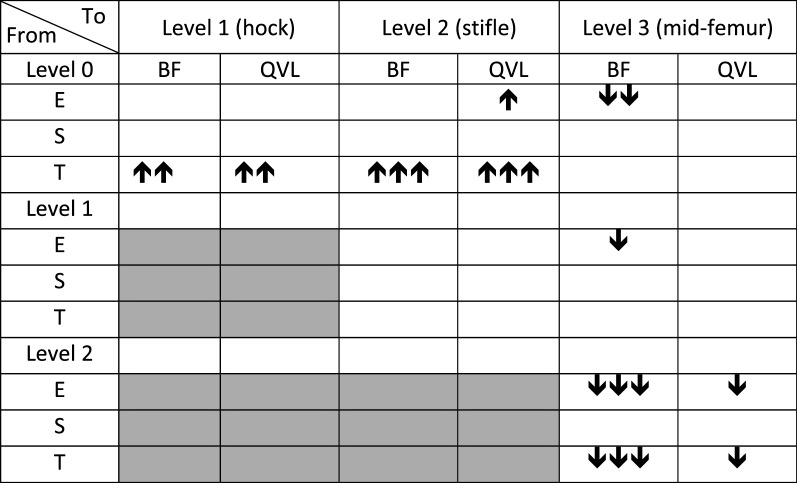
Changes in scores related to water level at the speed of 30 m/min

E-, S-, and T- scores of *m. biceps femoris* (BF) and *vastus lateralis of m. quadriceps* (QVL) obtained by acoustic myography of 25 dogs walking in a water treadmill. Water level 0: at the bottom of the pads. Increased (↑) or decreased (↓) scores are shown by 1, 2 or 3 arrows = P < 0.05, P < 0.01, P < 0.001, respectively

### Effects of water level at 50 m/min

The changes in E-, S- and T-scores that occurred with changing water levels at a fast walk are shown in Table 5. All scores for BF decreased when walking in water level 3 compared to a shallower depth. For QVL at this water level, only the E-scores decreased. At level 2 the T-scores for BF increased compared to walking in water level 0 or 1. However, when compared to level 1, this increase was counteracted by a decrease in the S-score.

**Table 5:**
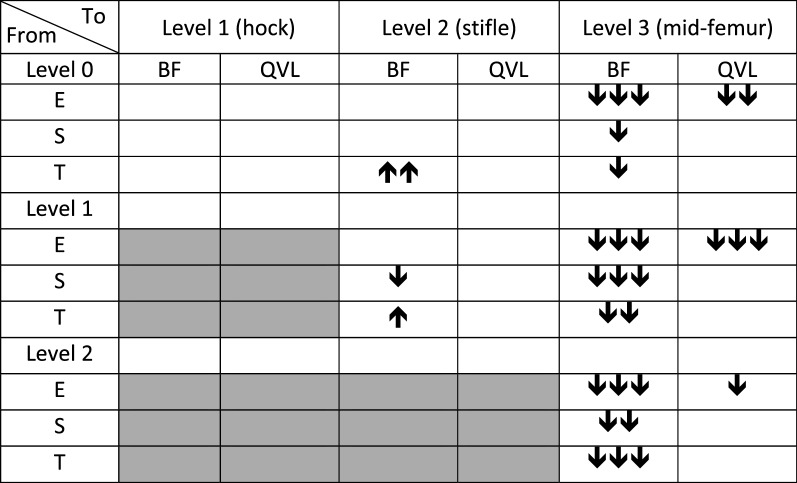
Changes in scores related to water level at the speed of 50 m/min

E-, S-, and T- scores of *m. biceps femoris* (BF) and *vastus lateralis of m. quadriceps* (QVL) obtained by acoustic myography of 25 dogs walking in a water treadmill. Water level 0: at the bottom of the pads. Increased (↑) or decreased (↓) scores are shown by 1, 2 or 3 arrows = P < 0.05, P < 0.01, P < 0.001, respectively

## Discussion

In support of the hypothesis, all AMG parameters (E-, S- and T-scores) decreased with increased speed for both muscles, showing an overall increased muscle activity at higher speed. In respect to the effect of water depth, the scores showed that a higher workload was required, when the dogs walked in a water depth at the level of mid-femur, compared to shallower water levels, and this finding was particularly evident for BF at a fast walk. An increase in workload with increasing speed and water heights up to the mid-thigh has also been documented by an increase in oxygen consumption in humans [5] and horses [6]. However, contrary to the afore-mentioned studies, in this study walking in hock- or stifle-deep water did not seem to increase workload of the two thigh muscles compared to no submersion. On the contrary, slow walking at water level 1 and 2 elicited a reduction in muscle firing frequency for both muscles compared to level 0, and this result was not compensated for by a higher recruitment of muscle fibers. Thus, walking slowly in water at the hock or stifle seemed to require less muscle activation in the tested muscles than walking in no water. It is possible that treadmill walking without water at a slow speed, especially when unaccustomed to this activity, requires more effort or skill than a faster speed, and because water slows movement [14], a balanced more natural gait is easier to achieve at 30 m/min in water. Still, also at the higher speed, scores at water level 1 or 2 did not indicate increased workload compared to less or no water; in fact, BF at water level 2 showed an elevation of the T-score, which was only partially counteracted by a decreased S-score. If extra muscle work is required to overcome the resistance of water, while the subject is submerged in water at the level of the mid-femur, as the scores indicate, it is surprising that lower water levels do not seem to increase muscle fiber activity compared to walking without water. The changes that occur in joint kinematics during aquatic exercise may offer a possible explanation. As a general rule, limb joint flexion increases when walking in water, and maximal flexion of the canine hip, stifle and hock is seen, when the water is at the level of the stifle [15]. This increased mobility may release elastic energy from tendons as outlined in the spring mass model [16], thereby lowering the demands for muscle contraction of the limbs. It is also important to note that the QVL and the cranial part of BF function primarily as extensors of the stifle joint, and increased workload that would be expected e.g. from stifle flexion in shallower water, would more likely be apparent in the caudal hamstring muscles. Although this study does not show increased muscle activity when walking in water below the mid-femur compared to no water, other muscles, e.g. limb flexors and core muscles, may be engaged in shallower water, resulting in an increase in total muscular workload, as suggested by the increased oxygen consumption documented in other species [5, 6]. A change in core muscle engagement has been shown in horses by an increased flexion of the pelvic back when walking in water [17, 18]. It is likely that the same is true for dogs, because back movement is linked to limb movement, and dogs, like horses, increase stride length in water [17, 19].

If the depth of water is higher than the mid-femur, the effect of buoyancy may lessen the muscular demands. Gleim & Nicholas [5] studied oxygen consumption and heart rate of men and women walking in a water treadmill at different depths. They found that both parameters increased with increasing water depth up to the mid-femur level, but decreased in waist-deep water. While oxygen consumption has not been studied in horses in deep water, Lindner et al. [1] found that heart rate was lower, when horses walked in a water level at 80% of the height of the withers, compared to shallower water. Buoyancy is likely to play a role here, but heart rate may also be influenced by changes in hydrostatic pressure as well as water temperature. Heart rate is known to decrease in cold water [2], and as horses, unlike dogs, are typically exercised in cool water, at a temperature around 15 °C [2, 15], one should probably be cautious about evaluating workload at different water levels based on alterations in the horses’ heart rate. Dogs tend to shift from walking to swimming, if the water level in the treadmill reaches above mid-femur, and therefore it would not only be difficult to conduct a study evaluating the workload associated with walking in deeper submersion than the mid-femur, but probably also of little practical relevance.

The findings of this study are not in alignment with a study by Parkinson and colleagues [4] that used sEMG to measure activity of the gluteus medius and the longissimus dorsi muscles on 8 dogs, while they walked in water. Recorded sEMG signals decreased, indicating less workload, when water was at mid-femur level compared to lower water levels. The gluteus medius is used to extend the hip of the dog as well as to assist with its medial rotation. It also serves to prevent lateral rotation of the hip during periods of load bearing. The BF likewise serves to extend the hip as well as the stifle joint, and the caudal part of the muscle serves to flex the stifle joint. Due to the similarities in function (hip extension/propulsion), one might expect the actions of these two muscles during the treadmill exercise to be similar. The discrepancy in activation reported by these two studies may be partly due to methodological differences. However, it is also likely that both sEMG and AMG will yield amplitudes for the BF that are significantly larger than for the gluteus medius, and therefore the effects of an intervention may be easier to detect reliably for the BF. This assumption is supported by a study by McLean et al. [20] that evaluated activation of BF, QVL and gluteus medius of dogs during various land-based physical exercises. Here the BF had the greatest sEMG amplitudes, and contrary to the other muscles, the amplitudes of the BF increased significantly during all given exercises compared to stance. It is possible that our results for BF were influenced by muscle activation from the surrounding musculature. Although we aimed at positioning the sensors over the cranial part of the BF, crosstalk from the caudal part of the BF as well as from other muscles, m. semitendinosus e.g., cannot be ruled out. Likewise, the results for the QVL may be influenced by crosstalk from the rectus femoris, which serves as a hip flexor as well as a stifle extensor.

Apart from crosstalk there are several other limitations to this study. With sensors attached to the skin there is a risk of signal interference by variations in skin contact, changes in the position of the skin relation to the underlying musculature, and variations in subcutaneous fat depositions. The data from our 25 dogs followed a uniform sequence-related pattern, indicating good signal quality and repeatability. Contrary to the Parkinson study [4], the dogs in this study showed no difference between right and left side measurements. Care was taken to avoid subtle gait disturbances by the recruitment of healthy active dogs, and all dogs were subjected to muscle palpation and gait analysis by an experienced veterinary surgeon prior to admittance in the study. Care was also taken to avoid distractions in the room during the trials, and the handler was always positioned in front of the treadmill. Proximity to water may present a possible challenge for analyses that require a stable contact of sensors to the skin. However, this factor may be less challenging with AMG than with sEMG as, unlike sEMG, the AMG signals have been shown not to be affected by human sweat [21]. With the sensor protected under the adhesive tape, proximity to water was not expected to interfere with the signals. Indeed, in the present study, acoustic gel was found between the skin and the sensor after the experiments, indicating that the water treadmill did not affect the skin/sensor interface.

Other limitations to consider are the lack of randomization and habituation. As the sequence of water levels was not randomized, it could be argued that the increased muscular demands shown at water level 3, being the last measured level in the sequence, could be a sign of muscle fatigue. However, the measurements at water level 0, first done at the beginning of the trial, were repeated immediately following level 3 measurements. When comparing the two level 0 results, there was no sign of fatigue, as none of the scores decreased at the second trial compared to the first [21]. On the contrary, the parameter for coordination (E-score) increased, when level 0 was performed at the end of the trial, indicating that the dogs were more at ease with moving on the treadmill during the later stage. And, presumably, the muscles were warmer at this stage; a factor that may affect the scores in accordance with a previous study where agility dogs were shown to have increased E-scores of their triceps brachii after a series of warm-up exercises [12]. Despite a possible effect of warm-up, the E-score was the score that was most consistently challenged for both muscles in water level 3. This indicates that the dogs had to engage more muscle power in order to stabilize the legs in deep water, which it is likely due to a more pronounced effect of buoyancy at this water level. Lack of habituation may be a factor here. The dogs were not trained for water walking, and it is not unlikely that with habituation E-scores might have been higher, as the dogs would have been more skilled at this kind of exercise.

The increased muscular demands seen in this study were primarily exerted on BF, while QVL was challenged to a much lesser degree. This can be explained by the increased demands on propulsive forces to overcome resistance which is highly influenced by water depth and speed [3]. Moreover, for dogs moving in a water treadmill, stance phase percentage is reduced with increasing water levels [19]. In high water, less weightbearing time together with load reduction due to buoyancy will reduce the demands on the QVL, being a weight bearing muscle, to a greater extent than the BF, which is a muscle engaged in propulsion.

Muscle strengthening is one of the rationales for including water treadmill exercise in a rehabilitation program, not least for post-surgical canine patients [22]. One common orthopedic disease in dogs is rupture of the cranial cruciate ligament (CCLR) [23], and the two muscles evaluated in this study are prone to extensive muscle atrophy following this condition despite surgical repair [22]. This study gives new knowledge on muscle activation that may help practitioners better targeting muscle build-up in the underwater treadmill. The applied walking speeds were also within a range that would likely be applicable to most large-breed CCLR patients at the strength-building stage of their rehabilitation. However, this study used only healthy dogs, and atrophied or weak muscles may be recruited in a different manner. For future research, a similar study performed in patients with muscle atrophy, after CCLR e.g., would be very useful.

As an assessor of muscle function, AMG has the advantage of being non-invasive and easy to use, and it does not involve pain or discomfort. Compared to sEMG, the analysis of data is more straight forward and with less question of interference from other signals [21], although the effects of crosstalk from underlying muscles now needs to be thoroughly investigated.

## Conclusion

Muscle strengthening is often a focus in canine rehabilitation. This study showed that the muscle activity required to walk in water at a depth above the stifle joint is greater than that required to walk at shallower water levels. Moreover, this exercise was found to be more demanding for the BF, a muscle engaged in propulsion, than for QVL. These findings provide valuable information that will help practitioners with the design of more precise protocols for water treadmill-based rehabilitation.

## Data Availability

The datasets used in the current study are available from the corresponding author on reasonable request.
